# Supplementary material for: Electromechanically reconfigurable optical nano-kirigami
Source: Nat Commun. 2021 Feb 26;12:1299. doi: 10.1038/s41467-021-21565-x (PMC7910307; doi:10.1038/s41467-021-21565-x)
Supplement: Supplementary file 1 — Supplementary Information [file 41467_2021_21565_MOESM1_ESM.pdf]

Supplementary Information for

Electromechanically reconfigurable optical nano-kirigami

Shanshan Chen[1,#], Zhiguang Liu[2,#], Huifeng Du[3,#], Chengchun Tang[2,#], Changyin Ji[1], Baogang Quan[2], Ruhao Pan[2], Lechen Yang[2], Xinhao Li[3], Changzhi Gu[2], Xiangdong Zhang[1], Yugui Yao[1], Junjie Li[2,*], Nicholas X. Fang[3,*], and Jiafang Li[1,2,*]

[1]*Key Lab of advanced optoelectronic quantum architecture and measurement (Ministry of Education), Beijing Key Lab of Nanophotonics & Ultrafine Optoelectronic Systems, and School of Physics, Beijing Institute of Technology, Beijing 100081, China*

[2]*Institute of Physics, Chinese Academy of Sciences, Beijing 100190, China*

[3]*Mechanical Engineering Department, Massachusetts Institute of Technology, Cambridge, Massachusetts 02139, USA*

The Supplementary Information include the following contents:

- **Supplementary Figure 1**. Geometric optimizations of nano-kirigami patterns.
- **Supplementary Note 1.** Discussions on Supplementary Figure 1.
- **Supplementary Figure 2**. Critical influence of the wet etching process.
- **Supplementary Figure 3**. Experimental demonstration of the significance of the geometric designs.
- **Supplementary Figure 4**. SEM images of various structural deformations induced by electrostatic forces.
- **Supplementary Figure 5**. Electrical tests and large-area samples.
- **Supplementary Figure 6.** Simulations on optical spectra.
- **Supplementary Figure 7**. Topography-dependent non-resonant optical modulations at visible wavelengths.
- **Supplementary Figure 8**. Reversible modulations of optical resonances at near-infrared wavelengths.
- **Supplementary Figure 9**. Reversible modulations of optical resonances under plastic deformations.
- **Supplementary Note 2.** Discussions on Supplementary Figure 9.
- **Supplementary Figure 10**. Discussions on the modulation of circular dichroism (CD).
- **Supplementary Note 3.** Details on the electromagnetic multipolar analysis in Supplementary Figure 10a.
- **Supplementary Figure 11.** Simulation results under oblique incidence.
- **Supplementary Figure 12**. Calculations on dynamic modulation properties.
- **Supplementary Note 4.** Discussions on time-domain responses in Supplementary Figure 12.

# These authors contributed equally to this work.

*Corresponding authors: jiafangli@bit.edu.cn, jjli@aphy.iphy.ac.cn, nicfang@mit.edu

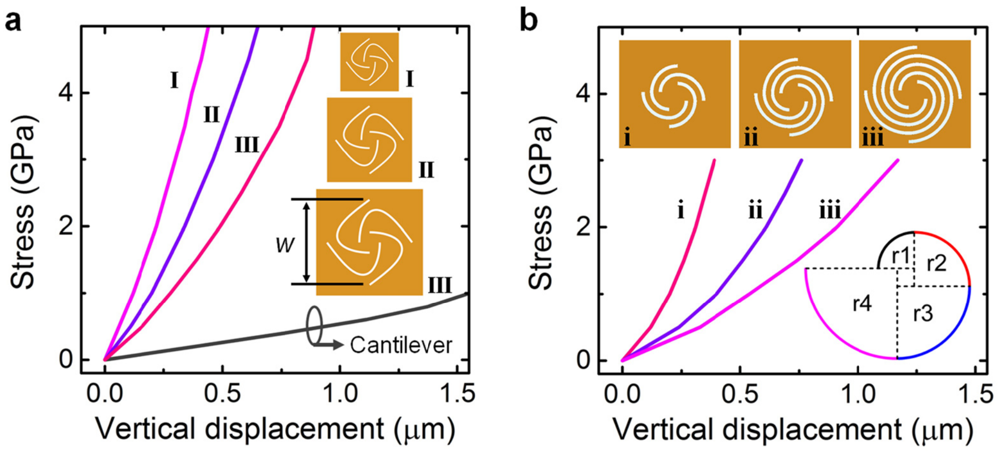

Supplementary Figure 1. Geometric optimizations of nano-kirigami patterns. Calculated relationship between pre-loaded stress and vertical displacement for different 2D patterns in the insets. **(a)** Pinwheels **I**, **II**, **III** and a cantilever with different widths. From **I** to **III**, the pinwheel widths are *w*=1.2, 1.6, 2 μm, respectively, of which the curve slopes are *S*=12.58, 7.79, and 5.52 GPa/μm, respectively. The square cantilever has a width of *w*=2 μm and slope of *S*=0.62 GPa/μm. **(b)** Type-i, -ii and -iii spiral structures consisting of four arms. Each arm consists of a serial of 90°-arc of different radius defined as $r(n)=(n+1)*0.175$, i.e., $r1=0.35$, $r2=0.525$, $r3=0.7$ and $r4=0.875$ μm, respectively, forming an Archimedean-like spiral, as shown in the inset. As shown in the top inset of **(b)**, the arc angles (lengths) of the spirals are **(i)** 180° (1.37 μm), **(ii)** 270° (2.47 μm) and **(iii)** 360° (3.85 μm), respectively, with *S*=7.69, 3.93 and 2.54 GPa/μm. From experimental experiences, for a design of $S\geq3.93$ GPa/μm, the structure can be freely suspended without collapse under the negative effects of the capillary forces.

Supplementary Note 1. Discussions on Supplementary Figure 1. According to the generalized Hooke's law $F=kz$ (k is the effective stiffness and z is the vertical displacement), the higher slope (S) of the curve in Supplementary Figure 1 indicates a larger effective stiffness k. Therefore, based on the effective mechanical frequency $\omega = \sqrt{k/m}$ [1], the large S of the pinwheels is more desirable for high-frequency operation than that of cantilevers with the same volume and mass. Moreover, it shows that the downscaling of the structure can help to further increase S (k) and decrease m (Supplementary Figure 1a). In such a way, the versatile 2D nano-kirigami patterns, as well as the downscaling effect, provide a convenient scheme for tailoring the electromechanical properties.

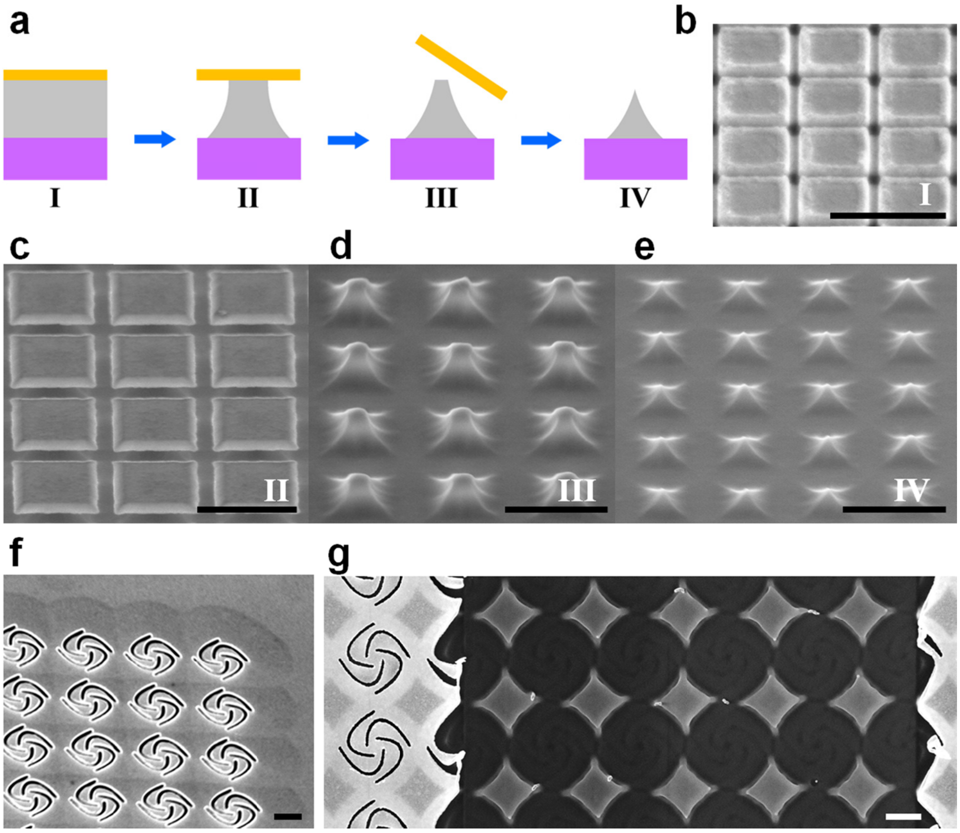

Supplementary Figure 2. Critical influence of the wet etching process. (a) Schematic of the shape evolution of the SiO_2 supporters under the wet etching. **(b,c,d,e)** SEM images of different meshes under different wet etching status, corresponding to the four typical conditions **I**, **II**, **III**, **IV** as noted in **(a)**. The shape of the SiO_2 supporters evolves from square frustums to pyramids with increased etching depth, which can be used as indicators for the controlling of etching speed. **(f)** SEM image of the gold pinwheel array collapsed onto the silicon substrate, which is caused by the over-etching of the SiO_2 layer. **(g)** Top-view SEM images of the pinwheel array, part of which has been lifted off by using high-dose irradiation of focused ion beam (FIB). Side-view SEM images of the same structure are shown in Fig. 2c. The SiO_2 supporters, in the shape of square frustums, are clearly seen. Scale bars: 1 μm.

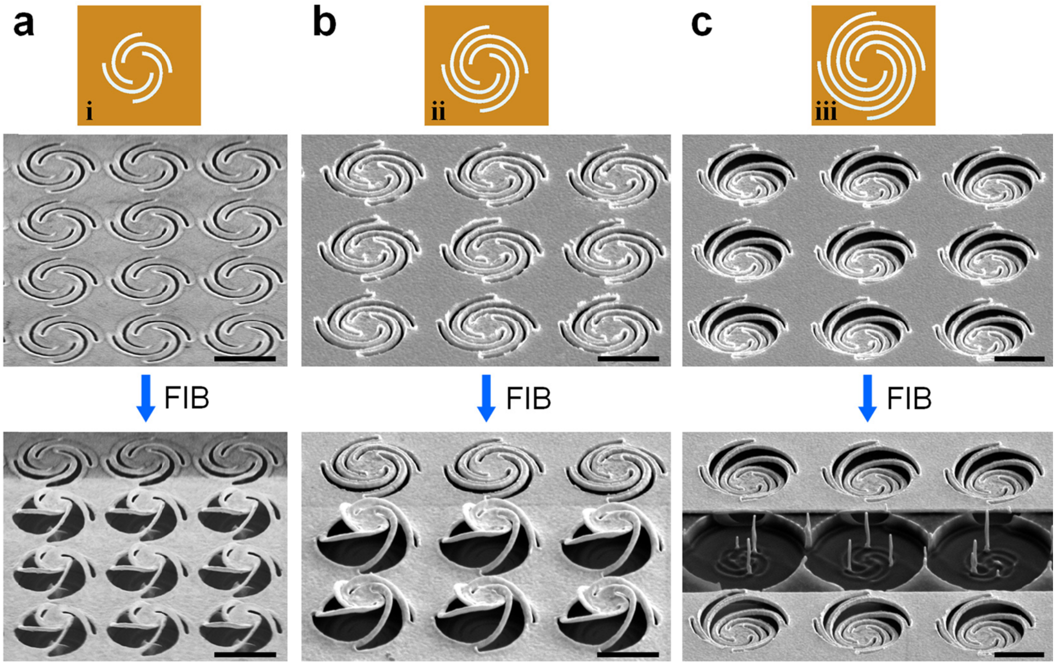

Supplementary Figure 3. Experimental demonstration of the significance of the geometric designs. (Top) Schematic and **(medium)** side-view SEM images of different combined spirals after wet etching. **(bottom)** SEM images of the part of spirals in the **(medium)** after FIB irradiation. The spiral structures in **(a,b,c)** correspond to the schematic spiral **i, ii, iii** in Supplementary Figure 1b, with arc angle (length) of **(i)** 180° (1.37 µm), **(ii)** 270° (2.47 µm) and **(iii)** 360° (3.85 µm), respectively. Compared to the type-i and type-ii spirals that are successfully suspended on the SiO_2 supporters, the type-iii spirals are collapsed under the capillary forces upon drying during the wet etching. As a result, the FIB irradiation induced stress can only bend the type-i/-ii spirals upward, while the type-iii spirals sticking to the bottom silicon are unable to be deformed under low-dose FIB irradiation. Nevertheless, the below SiO_2 supporters are uncovered after high-dose FIB radiation. Scale bars: 1 µm.

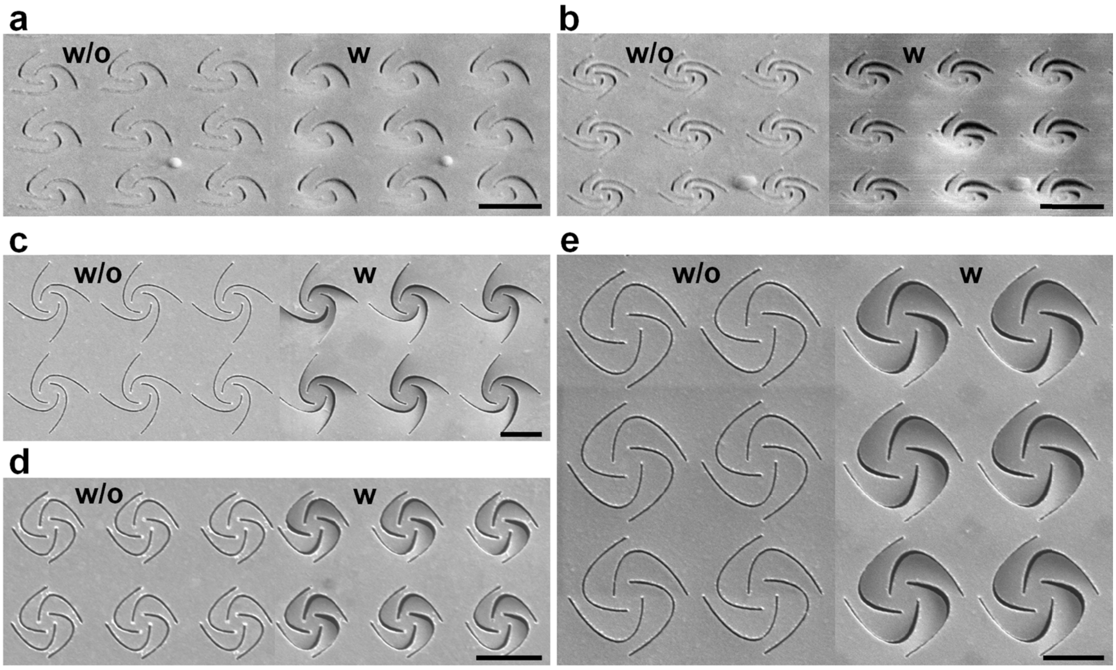

Supplementary Figure 4. SEM images of various structural deformations induced by electrostatic forces. (a,b) Side-view and **(c,d,e)** top-view SEM images of five kinds of nano-kirigami patterns without (**w/o**) and with (**w**) electrostatic forces induced deformations, showing clear downward deformations at all kinds and various scales. The width of the pinwheels in **(d)** and **(e)** is 1.1 and 2 μm, respectively. **(b)** is taken from Supplementary Movie 1. Scale bars: 1 μm.

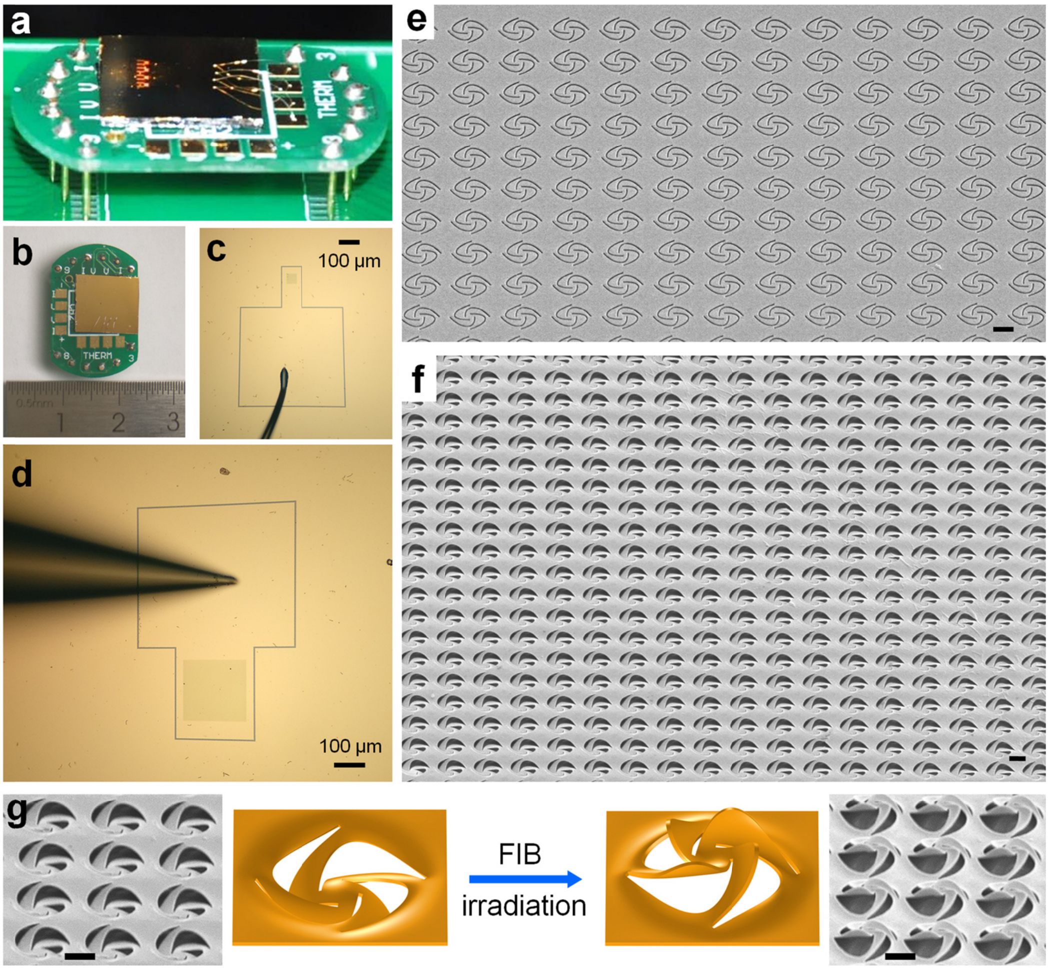

Supplementary Figure 5. Electrical tests and large-area samples. (a-d) Camera images of nano-kirigami structures fixed on electrical test boards under **(c)** wire bonding and **(d)** probe landing. The structural sizes are **(c)** 50×50 μm^2 and **(d)** 200×200 μm^2, respectively. **(e)** Side-view SEM images of an array of 2D pinwheels. **(f)** Side-view SEM images of an array of 3D deformed pinwheels after applying pull-in voltage. Size: 40×45 μm^2. **(g) (left)** Side-view SEM images and simulated images of the downward deformed pinwheels, and **(right)** their upward deformed counterparts after low-dose FIB irradiation. The experimental and calculated images are in good agreement. See Supplementary Movie 2 for continuous deformations. Scale bars in all SEM images: 1 μm.

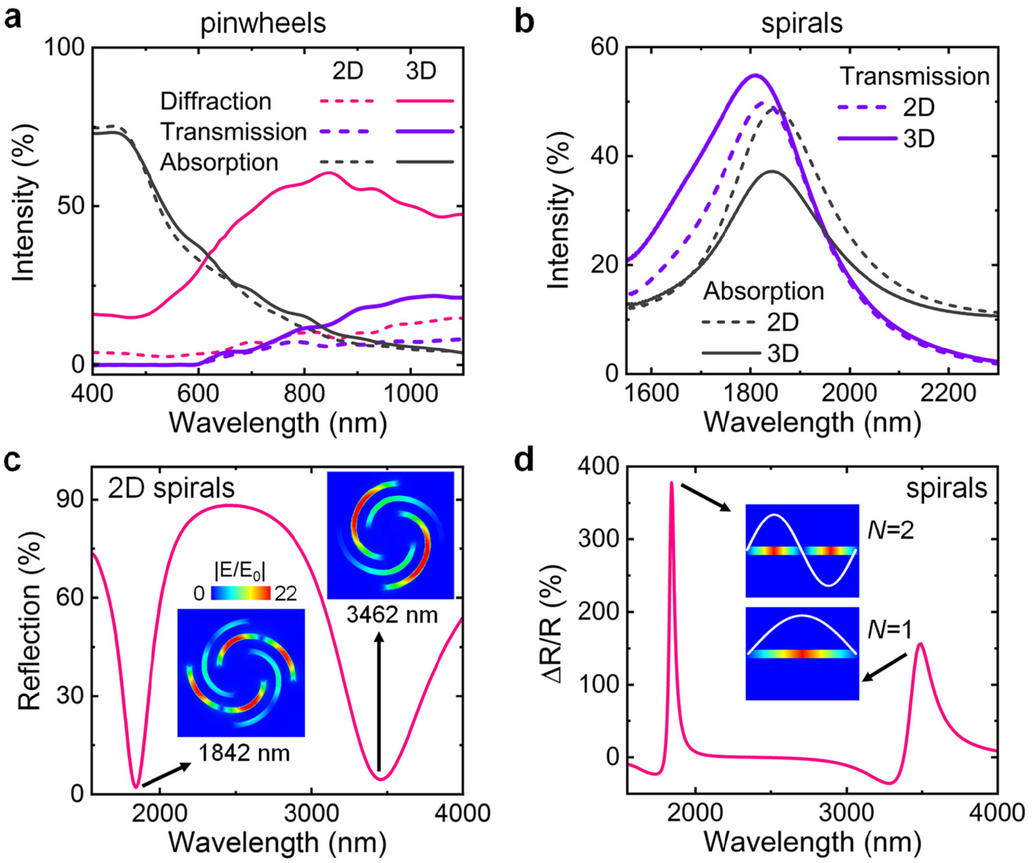

Supplementary Figure 6. Simulations on optical spectra. (a) Calculated transmission, absorption and high-order diffraction spectra of 2D and deformed 3D pinwheels (as in Fig. 3a, $\lambda \ll w$) under normal incidence. It can be seen that after the 3D deformations, the high-order diffraction to non-normal directions (red curves) is dramatically enhanced and the near-infrared transmission is slightly increased, while the change in absorption is negligible. **(b,c,d)** Calculated **(b)** transmission, absorption, **(c)** reflection spectra and **(d)** the associated modulation contrast of 2D and deformed 3D spirals (as in Fig. 3e, $\lambda > w$) under normal incidence. The 3D deformations induce enhanced transmission and reduced absorption in the near-infrared wavelengths. **Inset** of **(c)**, calculated electric field distributions of the 2D spirals in xy-plane (z=0) at λ=1842 and 3462 nm (corresponding to the two reflection dips). It can be seen that the air gaps of the spiral slits support strong plasmonic resonances that significantly enhance the light absorption and transmission, and thereby reduce the reflection at the two dips. In this case, the resonant wavelength λ is proportional to the equivalent length of the curved slit (here l_{slit}=1374 nm) by: $2n_{\mathrm{sp}}l_{\mathrm{slit}} = N\lambda$, where n_{sp} is the effective refractive index of the gap plasmons and N=1, 2, 3... **Inset** of **(d)**, schematic illustrations of the mode profiles near the modulation peaks with N=1 and 2, respectively.

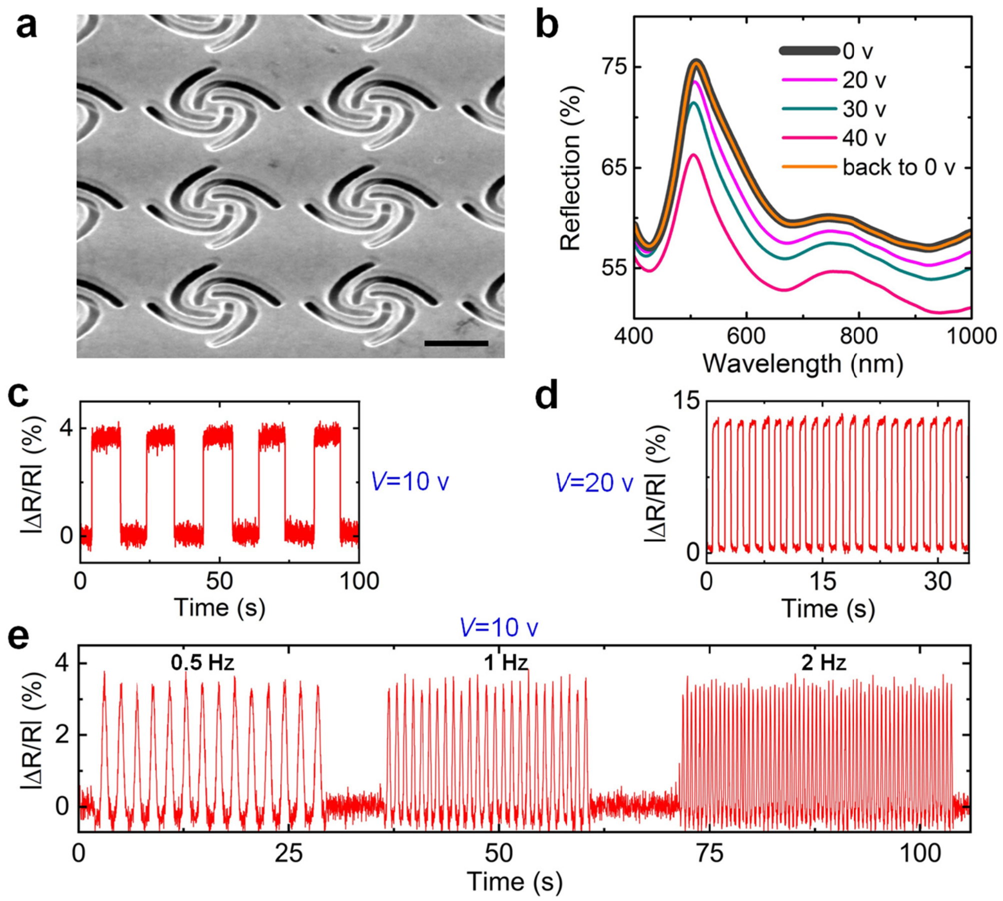

Supplementary Figure 7. Topography-dependent non-resonant optical modulations at visible wavelengths. (a) SEM image of a pinwheel with thinned arms. Structural parameters: w=2 μm, p=2.5 μm, d=300 nm. Scale bar: 1 μm. **(b)** Reflection spectra of the structure in **(a)** under different DC voltages as noted. After turning off the voltage at 40 v, the spectrum (orange) increases back to the initial 0 v condition, demonstrating the reversible optical modulation. **(c,d)** Modulation contrast at wavelength λ=550 nm versus time when the voltage is manually turned on and off for: **(c)** 5 cycles under V=10 v; **(d)** 20 cycles under V=20 v. **(e)** Modulation contrast under different frequencies as noted when the actuation voltage is controlled by a function signal generator under V=10 v and λ=550 nm. Here the manual and slow modulations were recorded by using a spectrometer (Ocean Optics, QE65000), while the fast modulations will be shown in following Supplementary Figure 8d. Together with Fig. 3 in the main text, the modulation properties are found dependent on both the geometric designs and fabricated linewidth of the initial 2D patterns.

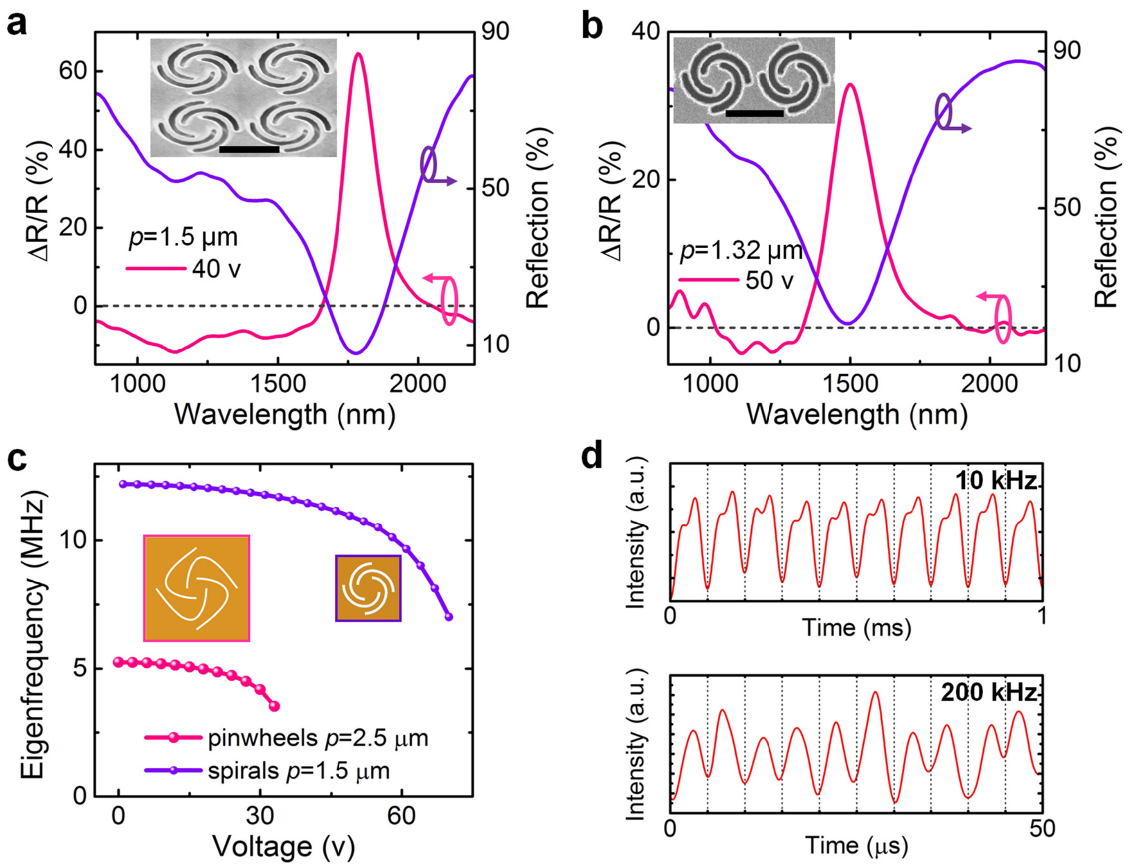

Supplementary Figure 8. Reversible modulations of optical resonances at near-infrared wavelengths. (a,b) (Left) Reversible modulation contrast in reflection versus wavelength under different DC voltages as noted and (**Right**) reflection spectra for the spirals with different periodicity shown in each **Inset**. The modulation turns back to zero instantly after the voltage is turned off, indicating elastic deformations are induced. Together with Fig. 3 in the main text, it can be seen that the reversible modulations are highly dependent on both the designed curves and fabricated linewidth. Scale bars: 1 μm. **(c)** Calculated mechanical eigenfrequency of the electromechanical pinwheels and spirals in Fig. 3 versus applied voltages. The operation frequency of the spirals (p=1.5 μm) can be over 10 MHz. **(d)** Preliminarily measured high-speed modulation of the reflected light intensity versus time at wavelength 1500 nm for the spirals in **(b)**. The modulation frequencies can reach **(top)** 10 kHz and **(bottom)** 200 kHz, respectively, under proper AC (alternating current) voltages, which are measured by using a fast photomultiplier tube (PMT, Hamamatsu, H10330A) and a lock-in amplifier (Zurich Instruments, HF2LI). The measurements are mixed with fluctuations in ambient atmosphere.

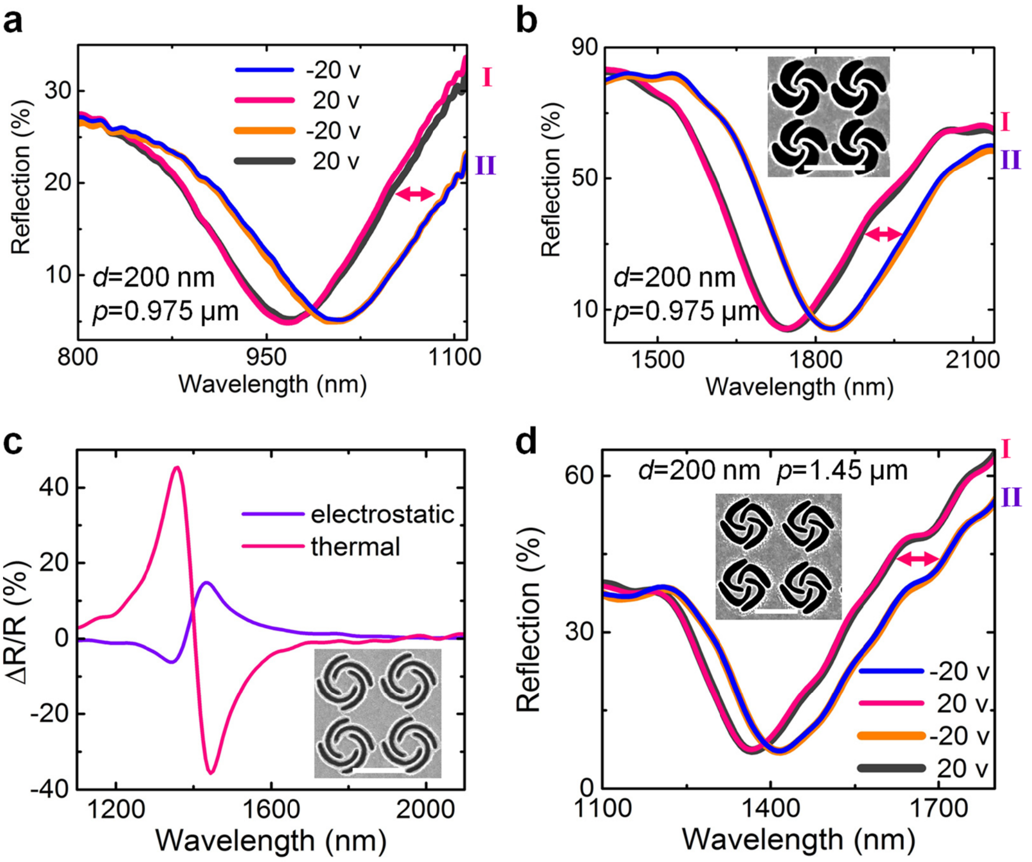

Supplementary Figure 9. Reversible modulations of optical resonances under plastic deformations. (a,b) Reflection spectra of the cross wires in the **Inset** of **(b)** at **State I** (*V*=20 v) and **State II** (*V*=-20 v), which are reversible with modulation contrast of 88% and 494% at wavelengths 953 and 1734 nm, respectively, as plotted in Fig. 4b. The two spectral regions correspond to the two modulation peaks in Fig. 4b. **(c)** Captured modulation contrast in reflection versus wavelength for a spiral structure in the inset, showing a spectral evolution from the blue lineshape under electrostatic force to the subsequent red lineshape under thermal effects. The modulation under electrostatic force results in the blue shift of the optical resonance, which is instant and can return to zero after turning off the voltage (also see in Fig. 3f). In comparison, the modulation by thermal effects induces the red shift of the optical resonance, which is slow and cannot return to zero after turning off the voltage due to the plastic deformations. As a result, the modulation spectra induced by the two different mechanisms show opposite lineshapes on the same structure. **(d)** Reflection spectra of the pinwheels in the **Inset** at two voltages as noted, showing two reversible states (**State I** and **State II**) as those in **(a-b)**, verifying the repeatability of the reversible modulations. Scale bars: 1 μm.

Supplementary Note 2. Discussions on Supplementary Figure 9. The slow and reversible modulations in Supplementary Figures 9a-9b result from the competition between the structural deformations induced by thermal expansion and electrostatic forces. First, the electrostatic forces pull the structures down into 3D, resulting in the blue shift of the spectra, as shown in Figs. 3e-3f of the main text. Second, when the SiO_2 layer is very thin and the applied voltage is high enough, the underlying leakage current induces thermal effects, which results in red-shift of the optical resonances due to the thermal expansion. The two effects induce opposite lineshapes in modulation spectra (as shown in Supplementary Figure 9c). The competition between the two effects forms a stable state under certain voltage. Meanwhile, due to the P-doped silicon substrate used in this work, the charge density at the space-charge region is much larger in the case of positive voltage than that under negative voltage. This causes stronger electrostatic force at V=20 v than that at V=-20 v, which results in the relatively shorter wavelengths of the optical resonances under V=20 v (as shown in Supplementary Figures 9a-9b and Fig. 4a). As a final result, the positive and negative voltages result in different spectral states, as shown in Supplementary Figures 9a-9b.

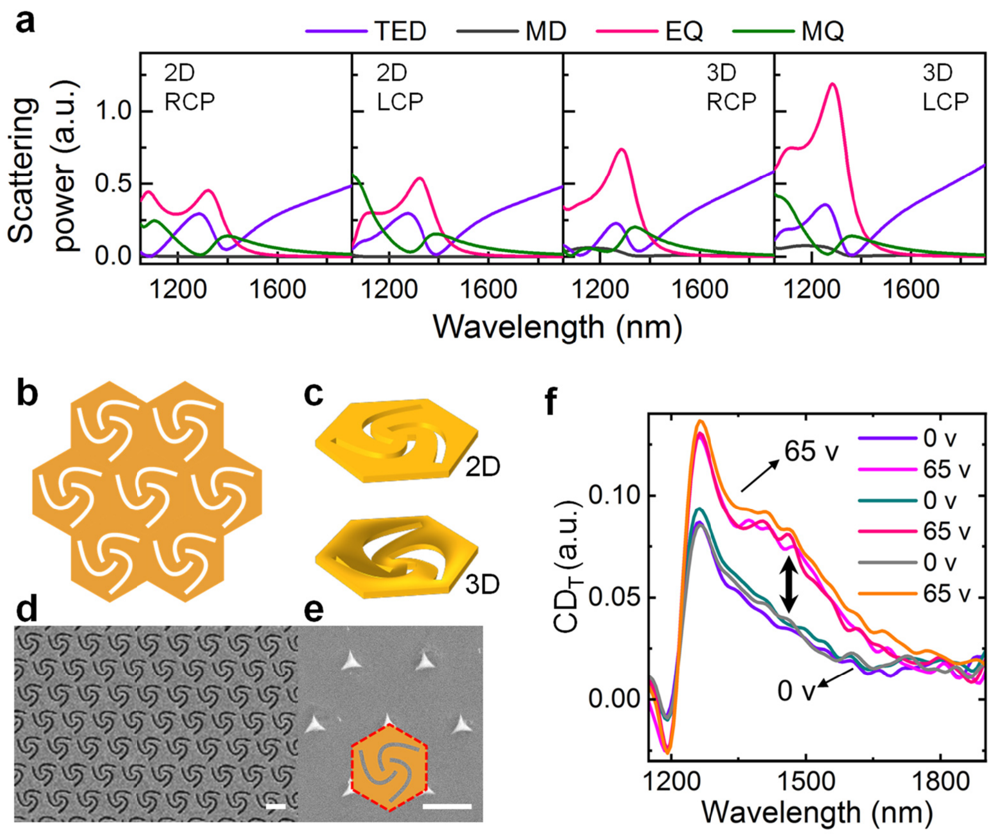

Supplementary Figure 10. Discussions on the modulation of circular dichroism (CD). (a) Calculated scattering power from various multipolar moments induced in the 2D and 3D three-arm pinwheels in Fig. 4c under RCP and LCP incidence, respectively. TED, the total electric and toroidal dipole moments; MD, magnetic dipole moment; EQ, electric quadruple moment; MQ, magnetic quadruple moment. The electromagnetic multipolar analysis was conducted by using the method in Ref. [2] (see below). The red spectral peaks of the 3D pinwheels indicate the handedness-dependent excitation of EQ modes, which induces the CD enhancement. **(b,c)** Schematic of the three-arm pinwheels in a hexagonal lattice. Here only the gold layers of the 2D and deformed 3D gold pinwheels are shown for clearness, which are employed in the numerical simulations of the inset in Fig. 4c. **(d,e)** Top-view SEM images of **(d)** fabricated three-arm pinwheels in a hexagonal lattice and **(e)** the below SiO_2 supporters after lifting-off the gold layer. The color image in (e) illustrates the relative position of the top pinwheel unit cell to the bottom triangular SiO_2 supporters. **(f)** Measured CD spectra of three-arm pinwheels with s=1.265 μm when the voltage is switched between 0 and 65 v. The CD strength is evaluated in transmission as CD_T (see *Methods*). It should be mentioned that the CD spectra of the three-arm pinwheels do not reach zero at V=0 v due to the slight plastic

deformations after a number of high-voltage tests. This result reveals the potentials in reversible electromechanical modulation of circular dichroism, which is promising and reserves spaces for further improvements. Scale bars: 1 μm.

Supplementary Note 3. Details on the electromagnetic multipolar analysis in Supplementary Figure 10a. The chiral responses were quantitatively analyzed by the decomposition of electromagnetic multipole moments excited by the LCP and RCP plane waves [2]. Specifically, the radiation power of total electric dipole (TED) moment, magnetic dipole (MD) moment, electric quadrupole (EQ) moment and magnetic quadrupole (MQ) moment based on the Cartesian multipole decomposition method can be calculated as $\sigma_{\mathrm{TED}} = \frac{\mu_0\omega^4}{12\pi c}|\mathbf{P} + \mathrm{ik}\mathbf{T}|^2$, $\sigma_{\mathrm{MD}} = \frac{\mu_0\omega^4}{12\pi c}|\mathbf{M}|^2$, $\sigma_{\mathrm{EQ}} = \frac{\mu_0\omega^4 k^2}{40\pi c}\sum_{\alpha,\beta}\left|\boldsymbol{Q}^E_{\alpha,\beta}\right|^2$, and $\sigma_{\mathrm{MQ}} = \frac{\mu_0\omega^4 k^2}{160\pi c}\sum_{\alpha,\beta}\left|\boldsymbol{Q}^M_{\alpha,\beta}\right|^2$. Here $\mathbf{P}$, $\mathbf{T}$, $\mathbf{M}$, $\boldsymbol{Q}^E_{\alpha,\beta}$, $\boldsymbol{Q}^M_{\alpha,\beta}$ are the electric dipole, toroidal dipole, magnetic dipole, components of electric and magnetic quadrupole moments, respectively, defined as

$$P_\alpha = \frac{1}{i\omega}\int d^3r\, J_\alpha(\mathbf{r}),$$

$$T_\alpha = \frac{1}{10c}\int d^3r\, \left[\left(\mathbf{r}\cdot\mathbf{J}(\mathbf{r})\right)r_\alpha - 2r^2 J_\alpha(\mathbf{r})\right],$$

$$M_\alpha = \frac{1}{2c}\int d^3r\, [\mathbf{r}\times\mathbf{J}(\mathbf{r})]_\alpha,$$

$$Q^E_{\alpha,\beta} = \frac{1}{2i\omega}\int d^3r\left[r_\alpha J_\beta(\mathbf{r}) + r_\beta J_\alpha(\mathbf{r}) - \frac{2}{3}\delta_{\alpha,\beta}\left(\mathbf{r}\cdot\mathbf{J}(\mathbf{r})\right)\right],$$

$$\text{and } Q^M_{\alpha,\beta} = \frac{1}{3c}\int d^3r\left[\left(\mathbf{r}\times\mathbf{J}(\mathbf{r})\right)_\alpha r_\beta + \left(\mathbf{r}\times\mathbf{J}(\mathbf{r})\right)_\beta r_\alpha\right],$$

where μ_0 is the permeability of vacuum, ω is the angular frequency, c is the speed of light in the vacuum, $\mathbf{r}$ specifies the location where the induced current is evaluated, and $\alpha, \beta = x, y, z$.

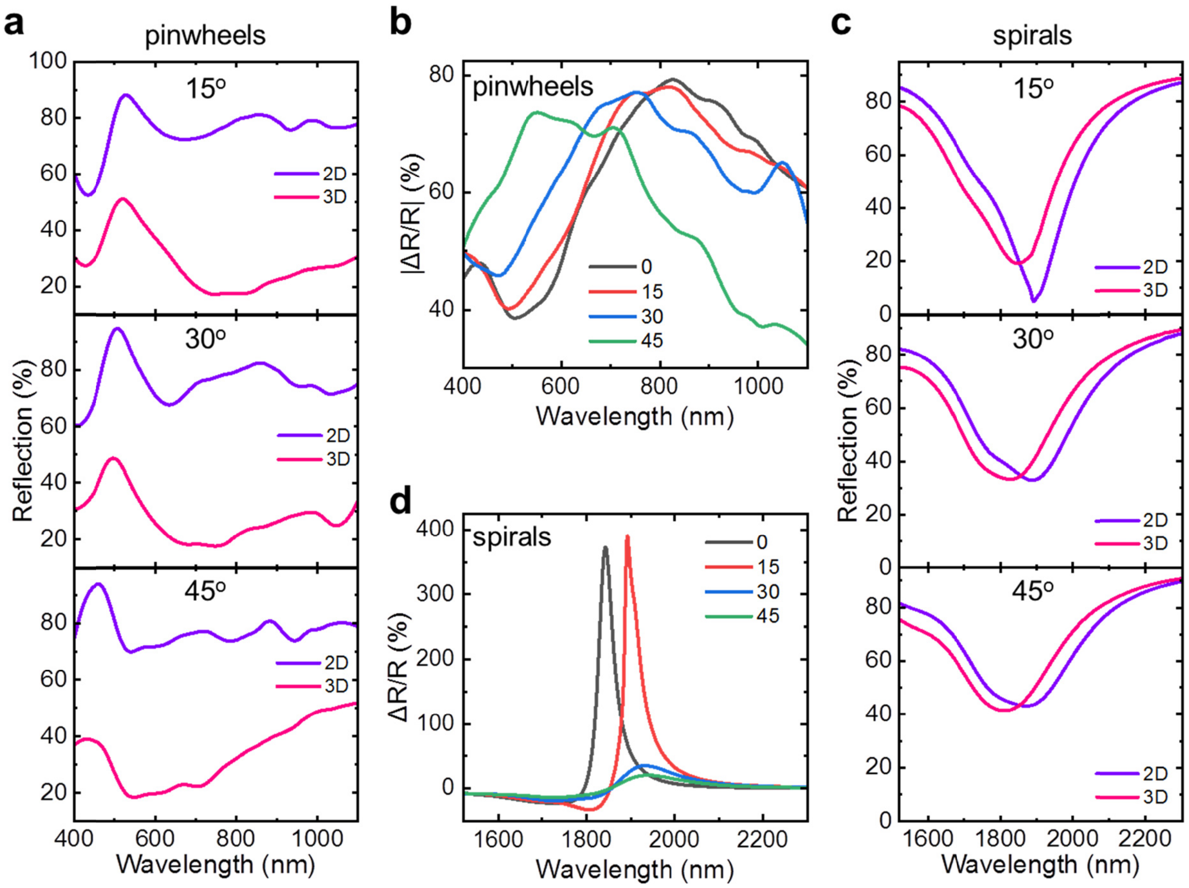

Supplementary Figure 11. Simulation results under oblique incidence. (a,c) Calculated reflection spectra of 2D and deformed 3D **(a)** four-arm pinwheels (as in Fig. 3a) and **(c)** spirals (as in Fig. 3e) under oblique incident angle of 15°, 30°, 45°, respectively. **(b)** Amplitude of modulation contrast (defined as $|\Delta R/R|$) versus wavelength for the pinwheels under different incident angle as noted. The non-resonant broadband modulation is blue shifted with the increase of incident angle, with maximum value slightly changed. **(d)** Modulation contrast versus wavelength for the spirals under different incident angle as noted. The resonant narrow-band modulation is slightly red shifted with the increase of incident angle, with peak value changed dramatically under large angles, which is caused by the angle-dependent excitation of plasmonic resonances.

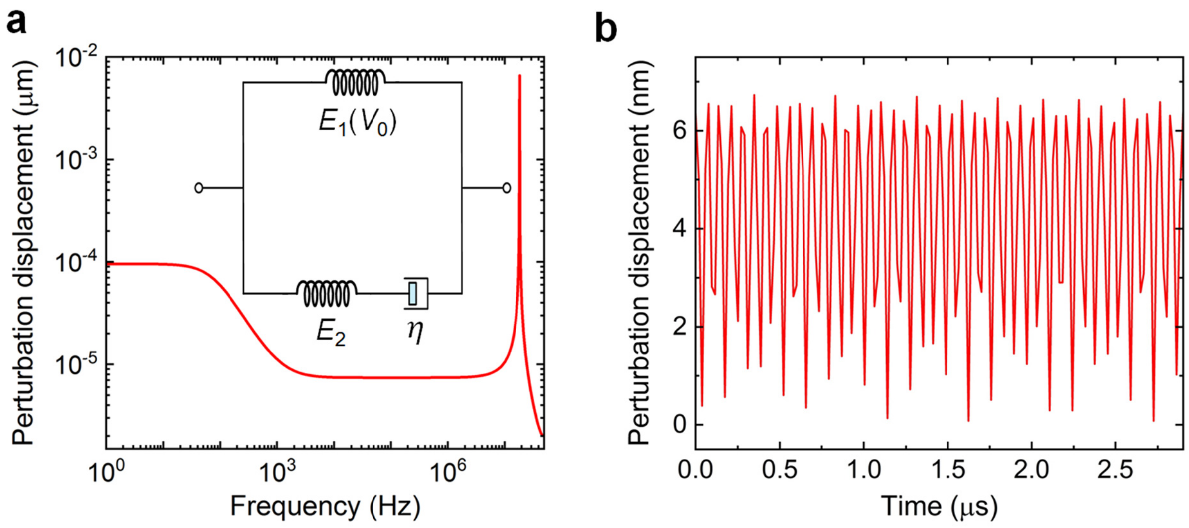

Supplementary Figure 12. Calculations on dynamic modulation properties. (a) Small-signal frequency response of the nano-kirigami structure (p=2.5 μm) at V_0=10 v biased position and an undulating amplitude of ΔV=0.1 v. The descending slope within the low-frequency regime is indicative of the hysteresis in structural response caused by the viscoelasticity of the material. The sharp peak corresponds to the resonant frequency of the damped system. **Inset**, schematic illustration of the Standard Linear Solid Model in calculations. $E_1(V_0)$ is the static modulus under the biased voltage V_0, and the lower branch is a combination of the elastic and viscous components, and $\eta = \tau E_2$, where τ is the relaxation time. **(b)** Vertical perturbation displacement versus time by applying frequency-to-time transformation of the response curve in **(a)** (see below discussions).

Supplementary Note 4. Discussions on time-domain responses in Supplementary Figure 12. Direct transient numerical analysis of high-frequency (above MHz) electro-actuated device are computationally challenging. This is because the strain field in the solids, velocity field of the air and electromagnetic fields in all metal-dielectric-fluid composites need to be solved simultaneously to simulate the coupled dynamics accurately [3-4]. In particular, the time complexity increases dramatically when the resonance frequency of structure exceeds the inverse of relaxation time of structure by orders of magnitude.

To bypass these challenges, we first computed the frequency response of the whole system within a wide range (Supplementary Figure 12a), ensuring both the low-frequency relaxation and high-frequency resonance were well-resolved. Subsequently, we performed

frequency-to-time transformation and derived the time response of the electromechanical nano-kirigami in Supplementary Figure 12b. Specifically, we demonstrated the feasibility of this approach by applying a Standard Linear Solid Model to characterize the viscoelastic behavior of materials under cyclic loading (as in the case of AC-actuated nano-kirigami schematically illustrated in the inset of Supplementary Figure 12a). Since any periodic load can be decomposed into the sum of biased DC voltages (V_0) and small-amplitude oscillating AC components (ΔV), the representation in the inset of Supplementary Figure 12a essentially serves as a characteristic eigenmode of the real motion of the system which would have been otherwise complicated to analyze. We also specified the material constants in this model in a more general way to include the dependence on the loading conditions. In this form of the model we chose $E_1 = 30\ \mathrm{GPa}$, $\tau = 0.0001\ \mathrm{s}$, $\eta = 2 \times 10^7\ \mathrm{Pa \cdot s}$ at a driving voltage composed of a bias of V_0=10 v and an undulating amplitude of ΔV=0.1 v. It should be mentioned that the introduction of the lower arm of spring and dashpot (in the inset of Supplementary Figure 12a) will increase the estimated resonant frequency of the system compared with the original result in Supplementary Figure 8c. Should the dissipations of the whole system (for example, material damping and air resistance) become negligible, the constitutive model (inset of Supplementary Figure 12a) would be reduced to the static limit where the upper arm alone determines the resonant frequency, as previously shown in Supplementary Figure 8c. We selected the center point of structure and monitored the variation of displacement as we swept through a wide frequency range (up to 60 MHz). The result is plotted in Supplementary Figure 12a. The decrease in deformation of structure below 10 kHz is due to the phase retardation between structural response and driving voltage. As it approaches the natural resonant frequency, the displacement amplitude also increases drastically, which corresponds to the peak of the response function in Supplementary Figure 12a.

After doing inverse fast Fourier transform, the time response of the system is plotted in Supplementary Figure 12b, where the nano-kirigami oscillates between two extreme points with a constant time interval, corresponding to the inverse of resonant frequency in Supplementary Figure 12a. We argue that this periodic response of the structural composite is analogous to the steady-state oscillation of the real system when the dissipation of energy is

compensated by the supply from the driving electrostatic force. The transition from initial system at rest to this steady state vibration requires a fully-coupled model from scratch with extremely high-resolved time steps and carefully-calibrated material dynamic properties, which will be the focus of our future study but is out of the scope of this work.

Supplementary References

[1] Midolo, L., Schliesser, A. & Fiore, A. Nano-opto-electro-mechanical systems. *Nat. Nanotechnol.* **13**, 11-18 (2018).

[2] Liu, Z. et al. Fano-enhanced circular dichroism in deformable stereo metasurfaces. *Adv. Mater.* **32**, 1907077 (2020).

[3] Chen, J. & Kang, S. Dynamic macromodeling of MEMS mirror devices. *IEEE International Electron Devices Meeting.* Cat. No. 01CH37224, 2001.

[4] Senturia, S.D., Aluru, N., & White, J. Simulating the behavior of MEMS devices: Computational methods and needs. *IEEE Computational Science and engineering* 4.1, 30-43 (1997).
