# Supplementary material for: Electromechanically reconfigurable optical nano-kirigami
Source: Nat Commun. 2021 Feb 26;12:1299. doi: 10.1038/s41467-021-21565-x (PMC7910307; doi:10.1038/s41467-021-21565-x)
Supplement: Supplementary file 3 — Description of Additional Supplementary Files [file 41467_2021_21565_MOESM3_ESM.docx]

**Description of Additional Supplementary Files**

**Supplementary Movie 1.** In-situ downward deformations of two pinwheel arrays attenuated by electrostatic forces induced by DC voltages under a scanning electron microscope (SEM).

**Supplementary Movie 2.** In-situ upward buckling of a pinwheel array induced by low-dose irradiation with focused ion beams (FIB) under a dual beam FIB/SEM system.
